# Dose-Dependent Incidence of Hepatic Tumors in Adult Mice following Perinatal Exposure to Bisphenol A

**DOI:** 10.1289/ehp.1307449

**Published:** 2014-02-03

**Authors:** Caren Weinhouse, Olivia S. Anderson, Ingrid L. Bergin, David J. Vandenbergh, Joseph P. Gyekis, Marc A. Dingman, Jingyun Yang, Dana C. Dolinoy

**Affiliations:** 1 Department of Environmental Health Sciences, and; 2 Unit for Laboratory Animal Medicine, University of Michigan, Ann Arbor, Michigan, USA; 3 Department of Biobehavioral Health,; 4 Neuroscience Program, and; 5 Methodology Center, Pennsylvania State University, University Park, Pennsylvania, USA; * These authors contributed equally to this work.

## Abstract

Background: Bisphenol A (BPA) is a high production volume chemical with hormone-like properties that has been implicated as a potential carcinogen. Early-life exposure has been linked to increased risk for precancerous lesions in mammary and prostate glands and the uterus, but no prior study has shown a significant association between BPA exposure and cancer development.

Objective: We explored the effects of BPA exposure during gestation and lactation on adult incidence of hepatic tumors in mice.

Methods: Isogenic mice were perinatally exposed to BPA through maternal diets containing one of four environmentally relevant doses of BPA (0, 50 ng, 50 μg, or 50 mg per kilogram of diet), and we followed approximately one male and one female per litter until they were 10 months of age. Animals were tested for known risk factors for hepatocellular carcinoma, including bacterial and viral infections.

Results: We found dose-dependent incidence of hepatic tumors in 10-month-old BPA-exposed mice. Of the offspring examined, 23% presented with hepatic tumors or preneoplastic lesions. We observed a statistically significant dose–response relationship, with an odds ratio for neoplastic and preneoplastic lesions of 7.23 (95% CI: 3.23, 16.17) for mice exposed to 50 mg BPA/kg diet compared with unexposed controls. Observed early disease onset, absence of bacterial or viral infection, and lack of characteristic sexual dimorphism in tumor incidence support a nonclassical etiology.

Conclusions: To our knowledge, this is the first report of a statistically significant association between BPA exposure and frank tumors in any organ. Our results link early-life exposure to BPA with the development of hepatic tumors in rodents, and have potential implications for human health and disease.

Citation: Weinhouse C, Anderson OS, Bergin IL, Vandenbergh DJ, Gyekis JP, Dingman MA, Yang J, Dolinoy DC. 2014. Dose-dependent incidence of hepatic tumors in adult mice following perinatal exposure to bisphenol A. Environ Health Perspect 122:485–491; http://dx.doi.org/10.1289/ehp.1307449

## Introduction

Bisphenol A (BPA) is an environmentally ubiquitous, high production volume chemical that has been linked to cardiovascular, immune, neuroendocrine, and reproductive end points (Diamanti-Kandarakis et al. 2009). Biomonitoring studies routinely detect levels of BPA in urine in > 90% of adults in the United States, indicating that exposure to BPA is widespread (Calafat et al. 2008). BPA has been classified as an endocrine disruptor and has been implicated in alterations in tissue enzyme and hormone receptor levels, interaction with hormone response systems, and cellular changes suggestive of carcinogenic potential (vom Saal et al. 2007).

The last large-scale evaluation of BPA’s potential carcinogenicity in multiple target organs was a National Toxicology Program (NTP) 2-year chronic feed study conducted in 1982, which employed doses ranging from 1,000 to 10,000 ppm BPA. Results provided inconclusive evidence for BPA’s carcinogenicity in the context of adult exposure. Nonsignificant incidences of liver tumors were reported in both sexes of rats and mice (NTP 1982). Subsequent early-life BPA exposure studies that examined mammary (Vandenberg et al. 2007) and prostate (Prins et al. 2008) glands and the uterus (Bergeron et al. 1999) reported precancerous lesions after perinatal exposure to BPA, but none have shown direct tumor development. Thus far, research on BPA and cancer has focused on reproductive and estrogen target organs, despite the ability of nonreproductive organs, such as the liver, to express estrogen receptors and respond to steroid hormone signaling (Cui et al. 2013). In the present study, we evaluated the effects of perinatal exposure to BPA at three environmentally relevant levels and observed dose-dependent incidence of hepatic tumors in adult mice at 10 months of age. To our knowledge, this is the first statistically significant report of clinically evident tumors—in addition to precancerous lesions—in any organ following perinatal or adult BPA exposure. Classically, male humans and rodents are two to four times as likely to develop hepatocellular carcinoma (HCC) as females (Hoenerhoff et al. 2011). Liver tumors are uncommon in rodents < 12 months of age and are often manifest at ≥ 20 months (Maronpot 2009). The combination of observed early disease onset and lack of characteristic sexual dimorphism in tumor incidence support a nonclassical etiology.

## Materials and Methods

*Animals and diets*. Mice were obtained from a colony that has been maintained with sibling mating and forced heterozygosity for the viable yellow *Agouti* (*A^vy^*) allele, resulting in a genetically invariant background (Waterland and Jirtle 2003). The *A^vy^* mutation initially arose spontaneously in C3H/HeJ mice; animals carrying the mutation were backcrossed with C57BL/6J mice, followed by > 220 generations of sibling mating (Waterland and Jirtle 2003). Based on these crosses, animals were calculated to be genetically 6.25–25% C3H/HeJ and 75–93.75% C57BL/6J (Waterland and Jirtle 2003). Observed rates of spontaneous or induced HCC in C57BL/6J mice have been reported as 2–10% (Maronpot 2009). As noted by Maronpot (2009), the C57BL/6J strain has been classified in numerous publications as “relatively resistant” to HCC. In the present study, the incidence rate we observed in our control animals is consistent with the reported rate in C57BL/6J mice.

Six-week-old virgin wild-type *a/a* female mice were randomly assigned to a phytoestrogen-free AIN-93G diet [TD.95092, with 7% corn oil instead of 7% soybean oil; Harlan Teklad, Madison, WI (diet composition is available online at http://www.harlan.com)] supplemented with one of four doses of BPA (0, 50 ng, 50 μg, or 50 mg/kg diet). The BPA was provided by the National Toxicology Program (NTP, Research Triangle Park, NC). Animals were housed in polycarbonate-free cages with *ad libitum* access to diet and BPA-free water.

After 2 weeks on the assigned BPA diet, 8-week-old virgin females were housed with *A^vy^/a* males and were impregnated within 0.5–5 days. Males were briefly exposed to diets containing BPA during the mating period.

This mating scheme produced approximately 50% wild-type (*a/a*) offspring and approximately 50% heterozygous (*A^vy^/a*) offspring. All pups were housed with their dams (which continued on their respective BPA diets) until weaning on postnatal day (PND) 22. The majority of *A^vy^/a* mice were euthanized at PND22 and analyzed for epigenetic changes, as previously reported (Anderson et al. 2012). A subset of wild-type (*a/a*) mice were then housed with a same-sex *A^vy^/a* sibling and fed the standard phytoestrogen-free control diet until 10 months of age, as described by Anderson et al (2013). Approximately 1 male and 1 female per litter for each of the BPA diets were chosen for inclusion in this subset: control (*n* = 10 males and 9 females), 50 ng/kg (*n* = 10 males and 10 females), 50 μg/kg (*n* = 10 males and 11 females), or 50 mg/kg (*n* = 9 males and 9 females).

In the present study, we analyzed the relationship between BPA exposure and hepatic tumor incidence in this subset of wild-type animals. This subset of offspring mice was previously assessed for metabolic and activity outcomes (Anderson et al. 2013).

We estimated that daily BPA exposure levels, based on a dam weighing 25 g and consuming 5 g chow daily, were 0, 10 ng, 10 μg, and 10 mg/kg body weight per day for diets containing BPA at 0, 50 ng, 50 μg, and 50 mg/kg of diet, respectively. We chose these diets to capture both mean and maximum human environmental exposures to BPA, which were reported by Vandenberg et al. (2013) to range from 0.1 to 5 μg/kg body weight per day. BPA exposure within human relevant ranges has been confirmed by direct measurement of BPA in liver tissue of a subset of exposed and control animals (Anderson et al. 2012). For example, total liver BPA measurements in animals fed the highest dose of BPA—50 mg/kg chow—ranged from 9.5 to 870 μg/kg liver; this includes the maximum human environmental exposure indicated by human fetal liver measurements, ranging from below the limit of quantitation (LOQ) to 96.8 μg BPA per kilogram of liver (Anderson et al. 2012). Anderson et al. (2012) reported BPA concentrations of < LOQ–11.3 μg/kg liver (mean 2 μg/kg; median 0.6 μg/kg) and < LOQ–13 μg/kg liver (mean 2.8 μg/kg; median 0.3 μg/kg) in livers from mice fed diets containing BPA at 50 μg/kg chow and 50 ng/kg chow, respectively. To prevent possible contamination with BPA from other sources, animals were singly housed in polypropylene cages (no polycarbonate plastics were used in animal management), and drinking water was tested once before the beginning of the exposure study by an independent, accredited public health and safety organization (NSF International, Ann Arbor, MI). Mice were housed in a facility approved by the Association for Assessment and Accreditation of Laboratory Animal Care (AAALAC) with a 12-hr light cycle, approximately 50% relative humidity, and temperature of 72 ± 2°C. Animals were maintained in accordance with the *Guide for the Care and Use of Laboratory Animals* (Institute of Laboratory Animal Resources 1996) and were treated humanely and with regard for alleviation of suffering. The study protocol was approved by the University of Michigan Committee on Use and Care of Animals.

*Histopathologic evaluation*. When mice were 10 months of age, liver tissue was collected, flash frozen in liquid nitrogen, fixed in formalin, and paraffin embedded. For each mouse, two or three slides containing liver sections, both with and without grossly visible masses, were evaluated for histopathology. Liver lesions were classified by light microscopy by an exposure-blinded, board-certified veterinary pathologist (I.L.B.), following recently revised, standardized guidelines established by the International Harmonization of Nomenclature and Diagnostic Criteria for Lesions in Rats and Mice (INHAND) project (Thoolen et al. 2010). This project represents consensus criteria for histopathological lesions in rodents as established by the North American, European, British, and Japanese Societies of Toxicologic Pathology. We did not clasify hyperplastic nodules as “regenerative” or “non-regenerative,” as specified by INHAND, because hepatopathic lesions, such as inflammation and oval cell hyperplasia, were present but no significant markers of liver injury, such as necrosis or fibrosis, were observed. Representative photomicrographs were taken using an Olympus DP72 12.5 megapixel digital camera mounted on an Olympus BX45 light microscope and using DP2-BSW software (all from Olympus Corporation of the Americas, Center Valley, PA). Photo processing and composite plate construction were performed using Adobe Photoshop CS4 (Adobe Systems, San Jose, CA). Images were acquired using the manufacturer’s software (CellSensStandard 1.7.1, Olympus Corporation), and image processing and composite construction was performed in Adobe Photoshop CS2, version 9.0.

*Bacterial and viral screens*. We used polymerase chain reaction (PCR) to determine whether liver tissue from eight animals from our colony (four at 10 months of age and four on PND22) were infected with *Helicobacter hepaticus* or *Helicobacter mastomyrinus*. PCR was performed using primers and positive controls from J.S. Opp and V. Young (University of Michigan) following the method of Eaton et al. (2011). The 10-month-old animals were included in the present study (one from each BPA diet group): two with hepatic tumors (one each from the 50 μg/kg and 50 mg/kg groups), and two without hepatic tumors [one from the control group (0 BPA) and one from the 50 ng group]. The remaining four animals (one from each BPA diet group) were siblings of the 10-month-old animals in the present study that were euthanized at PND22 (Anderson et al. 2012). The PCR analysis was performed on liver tissue of these eight animals at the end of the present study. We confirmed our animal selection and screening protocol with a board-certified veterinary pathologist (I.L.B.).

Serology testing for murine hepatitis virus (MHV) was performed via enzyme-linked immunosorbent assay (ELISA) (Wunderlich et al. 2011) every 6–8 weeks on sentinel animals that were not included in the present study. These sentinels (three animals per 50–70 cages of experimental animals) were housed in cages with small amounts of soiled bedding from randomly sampled experimental cages, and were changed once weekly. Sentinel animals were co-housed in our animal facilities throughout our exposure study.

*Single nucleotide polymorphism (SNP) genotyping*. Mice bred and raised at Penn State University, independent from the site for all other analyses, were used for genotyping. DNA was isolated from spleens of three male mice: one *A^vy^/a* and one wild-type *a/a*, both offspring of mice provided by R.L. Jirtle (Duke University), and one C57BL/6J purchased from The Jackson Laboratory (stock no. 000664; Bar Harbor, ME) as a 21-day-old weanling. These mice were maintained in an AAALAC-approved facility (12-hr light cycle, ~ 50% relative humidity, 72 ± 2°C) and fed LabDiet 5001 (Purina Inc., St. Louis, MO) in shoebox-style polycarbonate cages (27 cm × 15 cm × 13 cm) with corn cob bedding (Bed-O’ Cobs ¼®; Andersons Lab Bedding, Maumee, OH).

When the mice were 200 days of age, spleens were removed and the DNA isolated. After purification using a standard protocol of phenol/chloroform extraction followed by ethanol precipitation, the DNA was dissolved in water. The mice were genotyped by Geneseek (Neogen Corp., Lincoln, NE) on the Mega Mouse Universal Genotyping Array (MegaMUGA; Neogen Corp., Lansing, MI) for 74,800 microsatellite markers, spaced at approximately 33 KB intervals throughout the mouse genome. Data were processed in PLINK (v1.07; http://pngu.mgh.harvard.edu/~purcell/plink/) and SAS (v9.3; SAS Institute Inc., Cary, NC). Genotypes are available from the Mouse Phenome Database (MPD:484; http://phenome.jax.org).

*Statistical analysis*. Variables. We histologically identified 16 total tumors (benign and malignant), as well as four hyperplastic nodules, two of which co-occurred with tumors. Neoplastic and preneoplastic lesions were grouped in four different binary variables (present/absent): *a*) malignant HCCs only (*n* = 13); *b*) benign hepatic adenomas only (*n* = 3); *c*) all tumors combined (HCC and hepatic adenomas; *n* = 16); and (*d*) combined tumors and hyperplastic nodules (*n* = 18). Additional hepatic lesions analyzed as binary variables included steatosis, inflammation, Kupffer cell hyperplasia, oval cell hyperplasia, multinucleated hepatocytes, hepatocyte hypertrophy, and lipofuscin deposition. Total hepatic lesions (including tumors and all additional lesions listed) were evaluated as summary scores (1 point for presence of each lesion, summed across lesions). Because steatosis and inflammation may represent nonspecific background lesions whose inclusion may mask a true association, each score was tested in three ways: *a*) inclusion of all hepatic lesions; *b*) inclusion of all hepatic lesions except steatosis; and *c*) inclusion of all hepatic lesions except steatosis and inflammation. Associations between dose groups and hepatic lesions (9 variables) and summary scores (3 variables) were tested in the models below.

Models. To facilitate comparison of our data with those of the NTP carcinogenicity bioassay on BPA (NTP 1982), we used a nearly identical statistical strategy. We tested a total of 15 associations between BPA exposure level and hepatic lesion(s); all associations were tested using both exact tests and logistic regression models to account for bias inherent in each method, for a total of 30 models. Fisher’s exact tests and Cochran-Armitage exact tests of trend were used to detect associations *a*) between dose groups and hepatopathic lesions listed above, and *b*) between dose groups and trends in those lesions by dose, respectively. Fisher’s exact tests and Cochran-Armitage exact tests of trend were were run using the PROC FREQ statement with the EXACT option in SAS v9.3. Exact tests allow for conservative estimation of association significance given small cell counts, compared with potential overestimation of significance by chi-square tests of association; therefore, to prevent exacerbation of this bias, we stratified data by sex in exact test analyses only. We used logistic regression models, adjusted for clustering of mice within litters using generalized estimating equations (GEE), to test the same associations and trends. Poisson regression models, adjusted for clustering by litter, were run on summary score variables. Clustering prevents overestimation of association significance due to errant assumption of animal independence. Neither exact tests nor logistic regression models allow for simultaneous adjustment for small cell counts and litter; bias inherent in both methods tends to overestimate significance. Statistical significance was defined as *p* < 0.05 for all analyses. All statistical analyses were completed using SAS v9.3.

## Results

**Figure 1:**
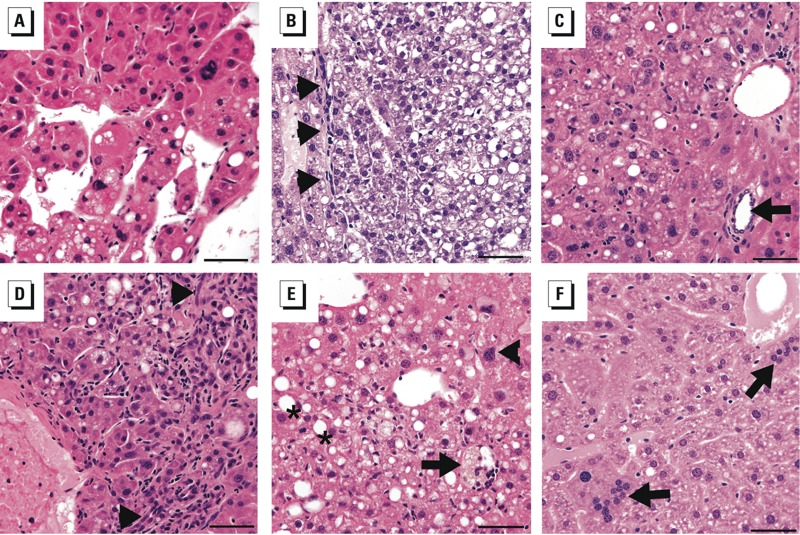
Representative photomicrographs of hematoxylin and eosin–stained hepatic lesions in wild-type (*a*/*a*) mice exposed to BPA through the maternal diet. (*A*) Hepatocellular carcinoma in a female mouse exposed to 50 mg BPA/kg maternal diet. (*B*) Hepatic adenoma in a male mouse exposed to 50 mg BPA/kg maternal diet; arrows indicate the line of demarcation between the neoplasm and compressed adjacent normal parenchyma. (*C*) Hyperplastic nodule in a female mouse exposed to 50 ng BPA/kg maternal diet; the arrow indicates a bile duct as part of a portal triad within the lesion, indicating preservation of hepatic architecture. (*D*) Oval cell hyperplasia (arrowheads) and increased Kupffer cells within sinusoids (Kupffer cell hyperplasia) in a male mouse exposed to 50 ng BPA/kg maternal diet. (*E*) Degenerative changes, including lipofuscin accumulation (arrow), hepatocellular hypertrophy (arrowhead), and steatosis (asterisks) in a female mouse exposed to 50 ng BPA/kg maternal diet. (*F*) Multinucleated hepatocytes (arrows) in a male mouse exposed to 50 mg BPA/kg maternal diet. Original magnification x400; bar = 50 μm.

**Figure 2:**
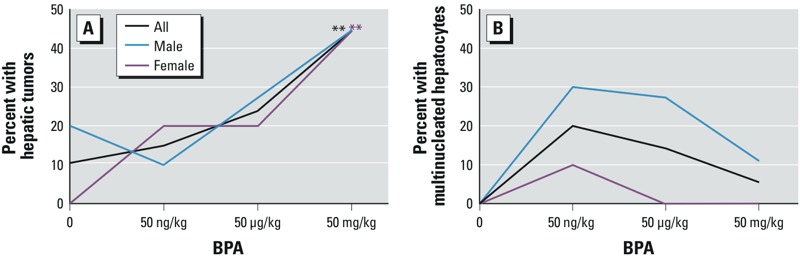
Incidence of hepatic tumors (*A*) or multinucleated hepatocytes (*B*) in wild-type (*a*/*a*) mice exposed perinatally to BPA through the maternal diet [0 (control), 50 ng, 50 μg, or 50 mg/kg of diet]. Mice exposed perinatally to BPA exhibited both linear and nonmonotonic dose responses in a lesion-specific manner. BPA-exposed mice exhibited (*A*) a statistically significant trend in hepatic tumors (neoplastic and preneoplastic hepatic lesions combined), and (*B*) a nonmonotonic trend in multinucleated hepatocytes, although this trend was not statistically significant. Numbers of animals per exposure group by lesion type and sex are given in Table 1. **p for trend < 0.05 by Cochrane-Armitage exact test of trend and logistic regression (see Supplemental Material, Tables S1 and S2).

**Figure 3:**
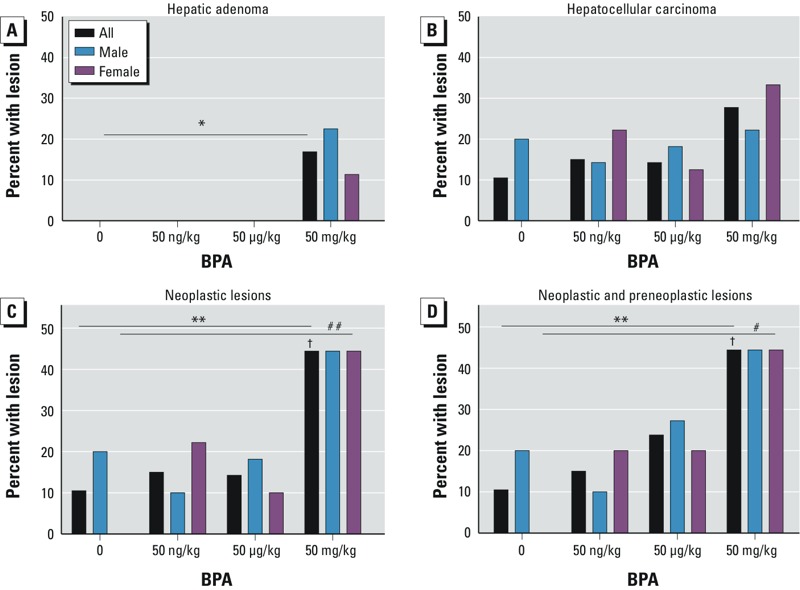
Dose-dependent incidence of hepatic tumors in wild-type (*a*/*a*) mice exposed perinatally to BPA through the maternal diet [0 (control), 50 ng, 50 μg, or 50 mg/kg of diet]. BPA-exposed mice exhibited (*A*) a statistically significant trend in hepatic adenomas (*n* = 3/78), (*B*) an increase in hepatocellular carcinomas (*n* = 13/78), (*C*) a statistically significant trend in neoplastic hepatic lesions (*n* = 16/78), and (*D*) a statistically significant trend in neoplastic and preneoplastic hepatic lesions (*n* = 18/78). Numbers of animals per exposure group by lesion type and sex are given in Table 1. For full statistical data, see Supplemental Tables S1 and S2. *p for trend < 0.05 by Cochrane-Armitage exact test of trend for total animals. **p for trend < 0.05 on both Cochrane-Armitage exact test of trend and logistic regression for total animals. ^#^*p* for trend < 0.05 on both Cochrane-Armitage exact test of trend and logistic regression for females only. ^##^*p* for trend < 0.1 for females only. ^†^*p* < 0.05 for OR by logistic regression.

*Histopathological evaluation*. During gestation and lactation, mice were exposed through maternal diet to one of four environmentally relevant doses of BPA (0, 50 ng, 50 μg, or 50 mg/kg of diet). We followed approximately one male and one female per litter until they reached 10 months of age (control, *n* = 19; 50 ng BPA, *n* = 20; 50 μg BPA, *n* = 21; and 50 mg BPA, *n* = 18). At dissection, 23.08% (*n* = 18/78) of offspring presented with neoplastic lesions (HCCs or hepatic adenomas) or preneoplastic lesions (hyperplastic nodules), with an odds ratio (OR) of 7.23 [95% confidence interval (CI): 3.23, 16.17; *p* = 0.014) for the 50 mg BPA group compared with controls. The dose response was significant on both Cochran-Armitage exact (*p* = 0.014) and logistic regression (*p* = 0.022) tests of trend (Figures 1A–C, 2, and 3, Table 1; see also Supplemental Material, Tables S1 and S2). Because murine hepatic adenomas and carcinomas are related pathologies (Hoenerhoff et al. 2011) and because preneoplastic lesions, including hyperplastic nodules, are often included in risk calculations for short-term carcinogenicity studies (Allen et al. 2004), we grouped benign adenomas, malignant carcinomas, and hyperplastic nodules for analysis. When preneoplastic lesions were excluded from the analysis (Figure 3; Table 1; see also Supplemental Material, Tables S1 and S2), results remained significant. Upon stratification by offspring sex, we observed a significant linear dose response for a combination of neoplastic and preneoplastic lesions in female animals (Figures 2A and 3D; see also Supplemental Material, Table S1). The presence of a statistically significant dose response in females but not in males does not necessarily indicate that the dose responses were significantly different between males and females.

**Table 1:** Percentage of hepatic lesions in wild-type *a*/*a* mice exposed perinatally to BPA at 0 (control), 50 ng, 50 μg, or 50 mg per kilogram of maternal diet, by dose and sex.

Hepatic lesion	BPA dose	Total mice	Males	Females
Hepatic adenoma	Total	3.85 (3/78)	5.00 (2/40)	2.63 (1/38)
	0	0 (0/19)	0 (0/10)	0 (0/9)
	50 ng	0 (0/20)	0 (0/10)	0 (0/10)
	50 μg	0 (0/21)	0 (0/11)	0 (0/10)
	50 mg	16.67 (3/18)	22.22 (2/9)	11.11 (1/9)
Hepatocellular carcinoma	Total	16.67 (13/78)	17.50 (7/40)	15.79 (6/38)
	0	10.53 (2/19)	20.00 (2/10)	0 (0/9)
	50 ng	15.00 (3/20)	14.29 (1/10)	22.22 (2/10)
	50 μg	14.29 (3/21)	18.18 (2/11)	12.50 (1/10)
	50 mg	27.78 (5/18)	22.22 (2/9)	33.33 (3/9)
Neoplastic lesions^*a*^	Total	20.51 (16/78)	22.50 (9/40)	18.42 (7/38)
	0	10.53 (2/19)	20.00 (2/10)	0 (0/9)
	50 ng	15.00 (3/20)	10.00 (1/10)	22.22 (2/10)
	50 μg	14.29 (3/21)	18.18 (2/11)	10.00 (1/10)
	50 mg	44.44 (8/18)	44.44 (4/9)	44.44 (4/9)
Neoplastic and preneoplastic lesions^*b*^	Total	23.08 (18/78)	25.00 (10/40)	21.05 (8/38)
	0	10.53 (2/19)	20.00 (2/10)	0 (0/9)
	50 ng	15.00 (3/20)	10.00 (1/10)	20.00 (2/10)
	50 μg	23.81 (5/21)	27.27 (3/11)	20.00 (2/10)
	50 mg	44.44(8/18)	44.44 (4/9)	44.44 (4/9)
Oval cell hyperplasia	Total	43.95 (34/78)	45.00 (18/40)	42.11 (16/38)
	0	26.32 (5/19)	40.00 (4/10)	11.11 (1/9)
	50 ng	30.00 (6/20)	20.00 (2/10)	40.00 (4/10)
	50 μg	66.67 (14/21)	72.73 (8/11)	60.00 (6/10)
	50 mg	50.00 (9/18)	44.44 (4/9)	55.56 (5/9)
Kupffer cell hyperplasia	Total	12.82 (10/78)	7.50 (3/40)	18.42 (7/38)
	0	15.79 (3/19)	20.00 (2/10)	11.11 (1/9)
	50 ng	15.00 (3/20)	0 (0/10)	30.00 (3/10)
	50 μg	9.52 (2/21)	9.09 (1/11)	10.00 (1/10)
	50 mg	11.11 (2/18)	0 (0/9)	22.22 (2/9)
Multinucleated hepatocytes	Total	10.26 (8/78)	17.50 (7/40)	2.63 (1/38)
	0	0 (0/19)	0 (0/10)	0 (0/9)
	50 ng	20.00 (4/20)	30.00 (3/10)	10.00 (1/10)
	50 μg	14.29 (3/21)	27.27 (3/11)	0 (0/10)
	50 mg	5.56 (1/18)	11.11 (1/9)	0 (0/9)
Steatosis	Total	50.00 (39/78)	47.50 (19/40)	52.63 (20/38)
	0	52.63 (10/19)	50.00 (5/10)	55.56 (5/9)
	50 ng	45.00 (9/20)	40.00 (4/10)	50.00 (5/10)
	50 μg	57.14 (12/21)	54.55 (6/11)	60.00 (6/10)
	50 mg	44.44 (8/18)	44.44 (4/9)	44.44 (4/9)
Inflammation	Total	50.00 (39/78)	42.50 (17/40)	57.89 (22/38)
	0	57.89 (11/19)	50.00 (5/10)	66.67 (6/9)
	50 ng	45.00 (9/20)	30.00 (3/10)	60.00 (6/10)
	50 μg	42.86 (9/21)	36.36 (4/11)	50.00 (5/10)
	50 mg	55.56 (10/18)	55.56 (5/9)	55.56 (5/9)
Hepatocyte hypertrophy	Total	32.05 (25/78)	27.50 (11/40)	36.84 (14/38)
	0	15.79 (3/19)	20.00 (2/10)	11.11 (1/9)
	50 ng	30.00 (6/20)	20.00 (2/10)	40.00 (4/10)
	50 μg	33.33 (7/21)	27.27 (3/11)	40.00 (4/10)
	50 mg	50.00 (9/18)	44.44 (4/9)	55.56 (5/9)
Lipofuscin deposition	Total	16.67 (13/78)	7.50 (3/40)	26.32 (10/38)
	0	5.26 (1/19)	0 (0/10)	11.11 (1/9)
	50 ng	15.00 (3/20)	0 (0/10)	30.00 (3/10)
	50 μg	23.81 (5/21)	18.18 (2/11)	30.00 (3/10)
	50 mg	22.22 (4/18)	11.11 (1/9)	33.33 (3/9)
All values are shown as percent (proportion). ^***a***^Defined as a combination of benign adenomas and malignant carcinomas. ^***b***^Includes adenomas, carcinomas, and preneoplastic nodules.				

**Figure 4:**
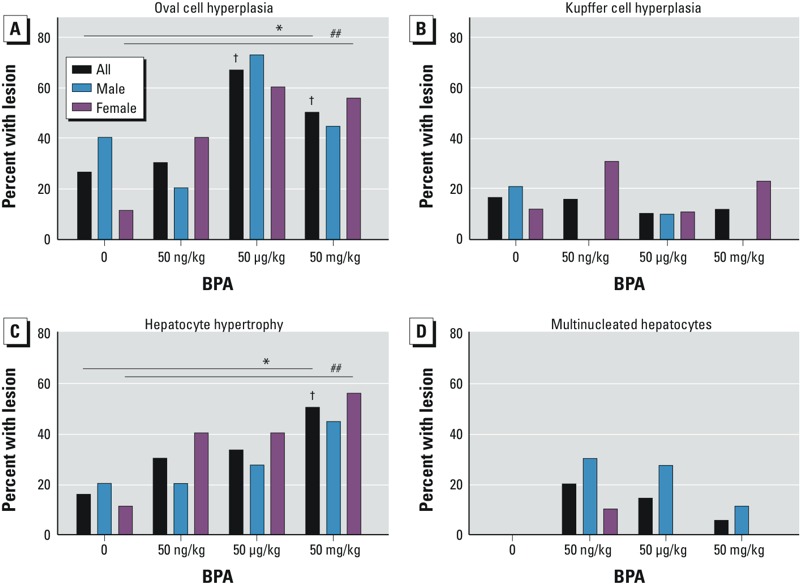
Dose-dependent incidence of proliferative lesions in wild-type (*a*/*a*) mice exposed perinatally to BPA through the maternal diet [0 (control), 50 ng, 50 μg, or 50 mg per kilogram of diet]. BPA-exposed mice exhibited (*A*) a statistically significant trend in oval cell hyperplasia, (*B*) no clear trend in Kupffer cell hyperplasia, (*C*) a statistically significant trend in hepatocyte hypertrophy, and (*D*) no clear trend in multinucleated hepatocytes. Numbers of animals per exposure group by lesion type and sex are given in Table 1. For full statistical data, see Supplemental Material, Tables S1 and S2. *p for trend < 0.05 on both Cochrane-Armitage exact test of trend and logistic regression for total animals. ^##^*p* for trend < 0.1 for females only. ^†^*p* < 0.05 for OR by logistic regression.

Almost half of the animals presented with oval cell or hepatobiliary stem cell hyperplasia (43.95%; *n* = 34/78), with significant ORs for the two highest dose groups [for 50 μg BPA, OR = 5.40 (95% CI: 3.26, 8.93), *p* = 0.001; for 50 mg BPA, OR = 2.67 (95% CI: 1.75, 4.06), *p* = 0.020)] (Figure 1D, Table 1; see also Supplemental Material, Tables S1 and S2). Approximately one-third of animals presented with hepatocyte hypertrophy (32.05%, *n* = 25/78), with a significant OR for the highest dose group (50 mg BPA): OR = 5.66 (95% CI: 2.57, 12.50), *p* = 0.028 (Figure 1E; Table 1; see also Supplemental Material, Tables S1 and S2). Incidences of oval cell hyperplasia and hepatocyte hypertrophy were significantly associated with increasing dose (Figure 4A,C; see also Supplemental Material, Tables S1 and S2). Animals with neoplastic lesions were significantly more likely to co-present with oval cell hyperplasia, hepatocyte hypertrophy, and Kupffer cell hyperplasia, suggesting a proliferative response to perinatal BPA exposure (see Supplemental Material, Tables S5 and S6). We observed multinucleated hepatocytes in 8 animals (10.26%, *n* = 8/78), primarily in males in low-dose groups, although the association with BPA exposure was not statistically significant (Figures 1F, 2B, and 4D, Table 1). Inflammation (50.00%; *n* = 39/78) and steatosis (50.00%; *n* = 39/78) may represent nonspecific markers of liver damage with age, rather than markers of chemical toxicity, because these lesions were distributed fairly uniformly across doses and controls, without any apparent pattern (Figure 1E, Table 1). Notably, no evidence of liver injury, such as fibrosis or necrosis, was present, suggesting that the proliferative lesions we observed were not a regenerative response to injury. When inflammation and steatosis were excluded from the analysis, the total number of hepatic lesions increased with dose; this indicates that hepatic lesions that were significantly associated with perinatal BPA exposure co-presented in the same animals (see Supplemental Material, Tables S3 and S4). Exposed dams did not present with any overt signs of obesity or other adverse health outcomes.

*Bacterial and viral screens*. To rule out known bacterial and viral disease risk factors, we performed a representative PCR screen for potential bacterial infection with *H. hepaticus* or *H. mastomyrinus* in liver tissue of exposed mice and assessed murine hepatitis viral load via serology measurements. Liver tissue from all animals evaluated tested negative on all bacterial and viral screens.

*SNP genotyping*. As determined previously by Waterland and Jirtle (2003), the mouse strain used in these experiments includes 6.25%–25% of the C3H/HeJ genome and 75%–93.75% of the C57BL/6J genome. C3H/HeJ mice are prone to spontaneous hepatocellular neoplasms and C57BL/6J mice are relatively resistant (Maronpot 2009). Up to 85% of this susceptibility of the C3H mouse to HCCs can be attributed to the *Hcs7* (hepatocarcinogenicity sensitivity 7) locus, located on the distal arm of chromosome 1 (Bilger et al. 2004; Drinkwater 1994). To empirically confirm the overlap between the genome of the mouse strain used in our experiments and that of the C57BL/6J strain, we genotyped 74,830 SNPs in two male mice derived from our colony and one male C57BL/6J mouse purchased from The Jackson Laboratory. Our strain’s genome differed from the C57BL/6J genome at a total of 5,247 SNPs and at only 6 of 5,416 SNPs on chromosome 1, indicating that our mice are genetically 93% C57BL/6J overall and > 99% C57BL/6J on chromosome 1 (see Supplemental Material, Table S7). Thus, our strain is genetically C57BL/6J at the *Hcs7* locus and, therefore, likely relatively resistant to spontaneous HCC.

## Discussion

In the present study, we observed dose-dependent incidence of hepatic tumors following perinatal exposure to BPA in an isogenic mouse model. Although mammary carcinomas have been reported in rodents perinatally exposed to BPA (Acevedo et al. 2013), the link was not statistically significant. To our knowledge, this is the first study to demonstrate a statistically significant relationship between BPA exposure and frank tumors of any reproductive or nonreproductive estrogen target organ. These tumors may be classified as early-onset disease because liver tumors are uncommon in all laboratory mouse strains at ages < 12 months, and are often evident at ≥ 20 months (Maronpot 2009). We did not note any apparent sexual dimorphism in disease incidence except in control animals. Classically, both male humans and rodents are two to four times as likely to develop HCC compared with females (Hoenerhoff et al. 2011). The combination of observed early disease onset and lack of characteristic sexual dimorphism in tumor incidence support a nonclassical etiology. These findings appear to be a function of dose and/or exposure timing; the adult rats and mice in the 1982 NTP carcinogenicity bioassay on BPA were exposed to doses estimated to be 20–200 times higher than the doses we used in the present study, but no significant increase in hepatic tumors was reported (NTP 1982).

Interestingly, like the NTP BPA bioassay (NTP 1982), we also observed dose-dependent incidence of multinucleated hepatocytes; however, the association between this lesion and BPA exposure was not statistically significant in either study. These abnormal cells may be found in aged mice, but they have been reported at younger ages in xenoestrogen-exposed rats and may be associated with increased hepatocyte proliferation (Hayashi et al. 2008; Scampini et al. 1993). Our data on increased multinucleated hepatocytes represent increased ploidy in mice without visible liver masses. BPA has previously been reported to induce meiotic aneuploidy in female mice (Hunt et al. 2003). Aneuploidy is the most common characteristic of solid tumors in humans (Kops et al. 2005). The presence of several proliferative lesions in exposed mice, including multinucleated hepatocytes and oval and Kupffer cell hyperplasia, in the absence of cellular necrosis or fibrosis, indicates an isolated proliferative response, and not a regenerative response following liver injury (Thoolen et al. 2010). Prior studies have noted a connection between BPA exposure and oxidative stress (Babu et al. 2013; Moon et al. 2012). Other studies have suggested that an exposure-mediated increase in reactive oxygen species (ROS) may have led to a concomitant increase in cellular proliferation in exposed mice via ROS signaling (Goodson et al. 2011; Hassan et al. 2012).

In the present study, animals tested negative on all bacterial and viral screens for infectious agents known to be promoters of HCC in rodents. As previously reported, gestational BPA exposure in these animals did not significantly influence litter size, survival, genotypic ratio, or sex ratio compared with control offspring (Anderson et al. 2012). Obesity and diabetes are well-documented risk factors for HCC in both rodents and humans. However, at 9 months of age, the exposed offspring examined in this study—regardless of tumor presence—exhibited body weights and serum glucose and insulin measurements at or below levels found in control animals (Anderson et al. 2013).

Because rodents have a high capacity for hepatocellular proliferation in response to liver damage, nongenotoxic factors may or may not be relevant to human exposures, although recent work suggests that molecular gene expression profiles of HCCs in B6C3F1 mice are similar to those of humans (Hoenerhoff et al. 2011). HCC is the sixth most common malignancy and the third most common cause of cancer-related deaths globally. Mortality rates in the United States are increasing more rapidly than for any other leading cancer, and age-adjusted incidence rates have doubled in the past 30 years, with an increase in early-onset disease in both sexes (Shaw and Shah 2011).

Although the majority (80%) of HCCs in humans can be attributed to hepatitis B virus or hepatitis C virus infection, further study of BPA’s role as a potential risk factor is warranted. Historically, use of first-generation oral contraceptives containing high doses of estradiol has been associated with increased rates of hepatic neoplasms, particularly hepatic adenomas (Giannitrapani et al. 2006). Recent studies have indicated that endogenous sex hormone levels can increase rates of carcinogenic conversion in individuals positive for hepatitis B virus (Wu et al. 2010). Ramirez et al. (2012) demonstrated that female rats given daily subcutaneous injections of 50 or 500 μg BPA (equivalent to 2.5–6.25 mg/kg BW and 25–62.5 mg/kg BW, respectively) from PND1 through PND10 experienced a loss of growth hormone–dependent sexual dimorphism in the liver’s ability to metabolize toxicants. Moon et al. (2012) found that intraperitoneal doses of 0.05–1.2 mg/kg BW/day administered to mice for 5 days induced hepatic mitochondrial dysfunction. In an epidemiological study of 1,455 adults, 18–74 years of age, Lang et al. (2008) observed a statistical association between increased urinary BPA concentration and clinically abnormal concentrations of the liver enzymes gamma-glutamyltransferase and alkaline phosphatase. Further, Betancourt et al. (2012) found that exposing lactating female rats to BPA at 250 μg/kg BW/day (estimated exposure to offspring, 2.5–25 ng BPA/kg BW/day) led to an increase in offspring susceptibility to subsequent chemical carcinogenesis.

Our study design has several notable strengths. We exposed mice to three doses that span several orders of magnitude, and the lower two doses are considered “low-dose” by two well-accepted definitions: a dose that does not exceed the threshold of the U.S. Environmental Protection Agency reference dose of 50 μg/kg BW/day and a dose that is within the range of observed human environmental exposure levels (Vandenberg et al. 2013). Our model was a well-accepted inbred rodent strain that is relatively resistant to the development of hepatic tumors. We exposed animals through the diet, which is currently accepted as a dominant route of exposure to BPA in humans (vom Saal et al. 2007). Animals were exposed during the perinatal period, capturing outcomes that may depend on exposure during critical developmental time points. Finally, we statistically clustered our data by litter, a method not used in many earlier BPA studies, which represents a significant criticism of and barrier to interpretation of prior studies.

A limitation of this study is the absence of direct maternal and fetal internal BPA dose measurements. However, comprehensive maternal and fetal measurements have been previously described. Zalko et al. (2003) determined that fetal free BPA levels peaked at approximately 4 ng/g 30 minutes after subcutaneous injection of pregnant CD-1 mice with BPA at 25 μg/kg BW, indicating that fetuses were exposed to approximately 6.25% of the administered dose. Sieli et al. (2011) found that bioavailability of BPA was higher in adult female C57BL/6J mice after dietary exposure to 100 mg BPA-d_6_/kg diet (similar to the present study’s maximum dose of 50 mg BPA/kg diet) compared with oral bolus administration, despite less efficient absorption of BPA through ingestion of food. In addition, mice exposed via diet exhibited higher maximum serum BPA concentrations and greater temporal delay in reaching maximum serum BPA concentrations than those receiving the oral bolus, indicating sustained circulating concentrations of BPA following dietary exposure (Sieli et al. 2011).

Our animal model and exposure scheme were initially chosen to evaluate the effects of perinatal BPA exposure on the mouse epigenome (Anderson et al. 2012) and on adult obesity risk (Anderson et al. 2013), rather than on liver tumorigenesis. However, SNP genotyping confirmed that our model is genetically similar to C57BL/6J mice at *Hcs7*, the locus reported to be associated with this strain’s resistance to HCC; thus, the mice we evaluated in this study represent a conservative model for liver cancer development. The limitations of this model are similar to those of any animal model: No direct conclusions can be drawn from this study on human health risk, particularly because human populations are genetically diverse and our model is isogenic. The use of an isogenic model, however, also represents a study strength, in that we were able to detect statistically significant outcomes without potentially confounding effects of individual differences in genetic susceptibility.

## Conclusions

To our knowledge, these data represent the first report of frank tumors in any organ following perinatal or adult BPA exposure in rodents. Our findings underscore the critical importance of exposure timing when evaluating adverse outcomes, particularly in light of nonsignificant liver tumor data in peripubertally exposed rodents in the NTP study (NTP 1982). These results implicate perinatal exposure to an environmentally ubiquitous chemical in the development of hepatic tumors in rodents, with potential implications for human health and disease.

## Supplemental Material

(339 KB) PDF
