# Supplementary material for: Dose-Dependent Incidence of Hepatic Tumors in Adult Mice following Perinatal Exposure to Bisphenol A
Source: Environ Health Perspect. 2014 Feb 3;122(5):485–91. doi: 10.1289/ehp.1307449 (PMC4014767; doi:10.1289/ehp.1307449)
Supplement: (339 KB) PDF [file ehp.1307449.s001.pdf]

Environmental Health Perspectives doi:10.1289/ehp.1307449

Supplemental Material

Dose-Dependent Incidence of Hepatic Tumors in Adult Mice following Perinatal Exposure to Bisphenol A

Caren Weinhouse, Olivia S. Anderson, Ingrid L. Bergin, David J. Vandenbergh, Joseph P. Gyekis, Marc A. Dingman, Jingyun Yang, and Dana C. Dolinoy

Table of Contents

Supplemental Material, Table S1. Exact tests of hepatic lesions by dose in mice exposed perinatally to BPA. 2

Supplemental Material, Table S2. Logistic regressions of hepatic lesions on dose in mice exposed perinatally to BPA. 3

Supplemental Material, Table S3. Total hepatic lesion scores in mice exposed perinatally to BPA. 5

Supplemental Material, Table S4. Logistic regressions of total hepatic lesion scores in mice exposed perinatally to BPA. 6

Supplemental Material, Table S5. Exact tests of associations between tumor status and hepatic lesions linked to cellular proliferation, in mice exposed perinatally to BPA. 7

Supplemental Material, Table S6. Logistic regressions of hepatic lesions linked to cellular proliferation on tumor status in mice exposed perinatally to BPA. 8

Supplemental Material, Table S7. SNP genotyping shows mice from this study are 93% C57BL/6J genome-wide, and >99% C57BL/6J on chromosome 1, including the *Hcs7* (*Hepatocellular carcinogenicity locus 7*) locus. 9

Supplemental Material, Table S1. Exact tests of hepatic lesions by dose in mice exposed perinatally to BPA. Fisher's exact tests and Cochran-Armitage exact tests of trend for hepatic lesions in mice exposed perinatally to control diet or to one of three doses of BPA (50 ng/kg diet, 50 µg/kg diet, or 50 mg/kg diet).

**Hepatic lesion**	**Group**	**Fisher's exact test (*p*-value)**	**Cochran-Armitage exact test of trend (*p*-value)**
Hepatic adenoma	Total	0.011	0.011
	Males	0.046	0.046
	Females	0.474	0.474
Hepatocellular carcinoma	Total	0.570	0.218
	Males	0.946	0.855
	Females	0.273	0.162
Neoplastic lesions	Total	0.061	0.021
	Males	0.384	0.236
	Females	0.089	0.056
Neoplastic and preneoplastic lesions[a]	Total	0.089	0.014
	Males	0.390	0.190
	Females	0.166	0.046
Oval cell hyperplasia	Total	0.036	0.037
	Males	0.115	0.392
	Females	0.133	0.052
Kupffer cell hyperplasia	Total	0.939	0.649
	Males	0.489	0.288
	Females	0.734	1.000
Multinucleated hepatocytes	Total	0.174	0.741
	Males	0.256	0.582
	Females	1.000	1.000
Steatosis	Total	0.840	0.918
	Males	0.972	1.000
	Females	0.970	0.884
Inflammation	Total	0.758	0.918
	Males	0.695	0.886
	Females	0.968	0.659
Hepatocyte hypertrophy	Total	0.178	0.036
	Males	0.642	0.267
	Females	0.277	0.094
Lipofuscin deposition	Total	0.384	0.131
	Males	0.486	0.193
	Females	0.740	0.410

[a]Neoplastic and pre-neoplastic lesions include adenomas, carcinomas, and pre-neoplastic nodules.

Supplemental Material, Table S2. Logistic regressions of hepatic lesions on dose in mice exposed perinatally to BPA. Odds ratios for hepatic lesions in mice exposed perinatally to one of three doses of BPA (control, 50 ng/kg diet, 50 µg/kg diet, or 50 mg/kg diet) were generated using logistic regression models, adjusted for clustering of mice within litters using Generalized Estimating Equations (GEE).

**Hepatic lesion**	**Dose (per kg diet)**	**Odds ratio (95% CI)**	**Parameter *p*-value**	***p* for trend**
Hepatocellular carcinoma	Control	Reference		
	50 ng BPA	1.36 (0.53, 3.53)	0.744	
	50 µg BPA	1.37 (0.70, 2.67)	0.643	
	50 mg BPA	3.01 (1.45, 6.27)	0.131	0.185
Neoplastic lesions	Control	Reference		
	50 ng BPA	1.49 (0.57, 3.86)	0.676	
	50 µg BPA	1.41 (0.70, 2.84)	0.620	
	50 mg BPA	6.75 (3.03, 15.00)	0.017	0.040
Neoplastic and preneoplastic lesions[a]	Control	Reference		
	50 ng BPA	1.59 (0.61, 4.12)	0.627	
	50 µg BPA	2.69 (1.27, 5.72)	0.189	
	50 mg BPA	7.23 (3.23, 16.17)	0.014	0.022
Oval cell hyperplasia	Control	Reference		
	50 ng BPA	1.15 (0.55, 2.41)	0.850	
	50 µg BPA	5.40 (3.26, 8.93)	0.001	
	50 mg BPA	2.67 (1.75, 4.06)	0.020	0.007
Kupffer cell hyperplasia	Control	Reference		
	50 ng BPA	1.27 (0.45, 3.60)	0.816	
	50 µg BPA	0.67 (0.25, 1.78)	0.679	
	50 mg BPA	0.68 (0.28, 1.66)	0.666	0.541
Steatosis	Control	Reference		
	50 ng BPA	1.07 (0.47, 2.49)	0.929	
	50 µg BPA	1.20 (0.55, 2.63)	0.815	
	50 mg BPA	1.11 (0.51, 2.43)	0.890	0.857
Inflammation	Control	Reference		
	50 ng BPA	0.59 (0.28, 1.26)	0.489	
	50 µg BPA	0.54 (0.32, 0.93)	0.254	
	50 mg BPA	0.90 (0.50, 1.63)	0.862	0.849
Hepatocyte hypertrophy	Control	Reference		
	50 ng BPA	2.40 (0.93, 6.22)	0.358	
	50 µg BPA	2.78 (1.33, 5.82)	0.165	
	50 mg BPA	5.66 (2.57, 12.50)	0.028	0.031
Lipofuscin deposition	Control	Reference		
	50 ng BPA	3.06 (0.98, 9.60)	0.328	
	50 µg BPA	5.47 (2.15, 13.93)	0.069	
	50 mg BPA	4.99 (1.81, 13.81)	0.114	0.087

[a]Neoplastic and pre-neoplastic lesions include adenomas, carcinomas, and pre-neoplastic nodules. Odds ratios for hepatic adenoma and multinucleated hepatocytes were not estimated, due to low lesion counts and subsequent lack of model convergence.

Supplemental Material, Table S3. Total hepatic lesion scores in mice exposed perinatally to BPA. Frequencies of co-occurring hepatic lesions in mice exposed perinatally to control diet or to one of three doses of BPA (50 ng/kg diet, 50 μg/kg diet, or 50 mg/kg diet), by dose. Values are shown as *percent of mice (proportion of mice)* presenting with a given number of lesions.

**Outcome and dose (per kg diet)**	**0 lesions**	**1 lesion**	**2 lesions**	**3 lesions**	**4 lesions**	**5 lesions**	**6 lesions**	**7 lesions**	**8 lesions**
Summary score (all lesions)[a]									
Control	36.84 (7/19)	10.53 (2/19)	21.05 (4/19)	10.53 (2/19)	5.26 (1/19)	15.79 (3/19)	0 (0/19)	0 (0/19)	0 (0/19)
50 ng BPA	25.00 (5/20)	35.00 (7/20)	15.00 (3/20)	0 (0/20)	0 (0/20)	10.00 (2/20)	5.00 (1/20)	5.00 (1/20)	5.00 (1/20)
50 μg BPA	0 (0/21)	42.86 (9/21)	14.29 (3/21)	4.76 (1/21)	9.52 (2/21)	0 (0/21)	19.05 (4/21)	4.76 (1/21)	0 (0/21)
50 mg BPA	16.67 (3/18)	27.78 (5/18)	11.11 (2/18)	5.56 (1/18)	5.56 (1/18)	0 (0/18)	33.33 (6/18)	0 (0/18)	0 (0/18)
Total	20.51 (15/78)	29.49 (23/78)	15.38 (12/78)	5.13 (4/78)	5.13 (4/78)	6.41 (5/78)	14.10 (11/78)	2.56 (2/78)	1.28 (1/78)
Summary score (less steatosis)									
Control	42.11 (8/19)	26.32 (5/19)	10.53 (2/19)	5.26 (1/19)	10.53 (2/19)	5.26 (1/19)	0 (0/19)	0 (0/19)	-
50 ng BPA	40.00 (8/20)	25.00 (5/20)	10.00 (2/20)	0 (0/20)	10.00 (2/20)	5.00 (1/20)	5.00 (1/20)	5.00 (1/20)	-
50 μg BPA	19.05 (4/21)	38.10 (8/21)	4.76 (1/21)	14.29 (3/21)	0 (0/21)	19.05 (4/21)	4.76 (1/21)	0 (0/21)	-
50 mg BPA	27.78 (5/18)	16.67 (3/18)	16.67 (3/18)	0 (0/18)	5.56 (1/18)	27.78 (5/18)	5.56 (1/18)	0 (0/18)	-
Total	32.05 (25/78)	26.92 (21/78)	10.26 (8/78)	5.13 (4/78)	6.41 (5/78)	14.10 (11/78)	3.85 (3/78)	1.28 (1/78)	-
Summary score (less steatosis and inflammation)									
Control	68.42 (13/19)	10.53 (2/19)	5.26 (1/19)	10.53 (2/19)	5.26 (1/19)	0 (0/19)	0 (0/19)	-	-
50 ng BPA	55.00 (11/20)	15.00 (3/20)	5.00 (1/20)	10.00 (2/20)	5.00 (1/20)	5.00 (1/20)	5.00 (1/20)	-	-
50 μg BPA	23.81 (5/21)	33.33 (7/21)	19.05 (4/21)	0 (0/21)	19.05 (4/21)	4.76 (1/21)	0 (0/21)	-	-
50 mg BPA	38.89 (7/18)	11.11 (2/18)	11.11 (2/18)	5.56 (1/18)	27.78 (5/18)	5.56 (1/18)	0 (0/18)	-	-
Total	46.15 (36/78)	17.95 (14/78)	10.26 (8/78)	6.41 (5/78)	14.10 (11/78)	3.85 (3/78)	1.28 (1/78)	-	-

[a]Total summary scores included the following ten lesions: hepatic adenoma; hepatocellular carcinoma; hyperplastic nodule; oval cell hyperplasia; Kupffer cell hyperplasia; multinucleated hepatocytes; steatosis; inflammation; hepatocyte hypertrophy; lipofuscin deposition. No animal presented with greater than eight (8) lesions. Two additional scores were computed, excluding steatosis or both steatosis and inflammation, to avoid masking true effects with highly prevalent background lesions.

Supplemental Material, Table S4. Logistic regressions of total hepatic lesion scores in mice exposed perinatally to BPA. Odds ratios for total, co-occurring hepatic lesions in mice exposed perinatally to control diet or to one of three doses of BPA (50 ng/kg diet, 50 µg/kg diet, or 50 mg/kg diet) were generated using logistic regression models, adjusted for clustering of mice within litters using Generalized Estimating Equations (GEE).

Outcome and dose (per kg diet)	Odds ratio (95% CI)	Parameter *p*-value	*p* for trend
Summary score (all lesions)[a]			
Control	Reference		
50 ng BPA	1.30 (0.83, 2.06)	0.558	
50 µg BPA	1.56 (1.19, 2.04)	0.097	
50 mg BPA	1.76 (1.28, 2.41)	0.075	0.086
Summary score (less steatosis)	Reference		
Control	1.40 (0.85, 2.30)	0.496	
50 ng BPA	1.69 (1.24, 2.28)	0.073	
50 µg BPA	1.98 (1.40, 2.79)	0.046	0.054
50 mg BPA			
Summary score (less steatosis and inflammation)			
Control	Reference		
50 ng BPA	1.85 (1.03, 3.30)	0.293	
50 µg BPA	2.41 (1.62, 3.58)	0.026	
50 mg BPA	2.70 (1.74, 4.18)	0.024	0.023

[a]Total summary scores included the following ten lesions: hepatic adenoma; hepatocellular carcinoma; hyperplastic nodule; oval cell hyperplasia; Kupffer cell hyperplasia; multinucleated hepatocytes; steatosis; inflammation; hepatocyte hypertrophy; lipofuscin deposition. No animal presented with greater than eight (8) lesions. Two additional summary scores were computed, excluding steatosis or excluding both steatosis and inflammation, to avoid masking true effects with highly prevalent background lesions.

Supplemental Material, Table S5. Exact tests of associations between tumor status and hepatic lesions linked to cellular proliferation, in mice exposed perinatally to BPA. Fisher's exact tests for associations between tumor status and hepatic lesions linked to cellular proliferation, in mice exposed perinatally to control diet or to one of three doses of BPA (50 ng/kg diet, 50 µg/kg diet, or 50 mg/kg diet), stratified by sex. Lesion percents and proportions are shown as the fraction of animals presenting with the lesion of interest among animals with neoplasms and among animals without neoplasms.

Hepatic lesion	Group	Neoplastic lesion	No neoplastic lesion	Fisher's exact test (*p*-value)
Oval cell hyperplasia	Total	93.75 (15/16)	30.65 (19/62)	5E-6
	Males	88.89 (8/9)		0.006
	Females	100.00 (7/7)		9E-4
Kupffer cell hyperplasia	Total	37.50 (6/16)	6.45 (4/62)	0.004
	Males	22.22 (2/9)		0.122
	Females	57.14 (4/7)		0.013
Hepatocyte hypertrophy	Total	93.75 (15/16)	16.13 (10/62)	1E-8
	Males	88.89 (8/9)		2E-5
	Females	100.00 (7/7)		3E-4

Neoplastic lesions include hepatocellular carcinomas and hepatic adenomas. Multinucleated hepatocytes may be associated with hepatocyte proliferation but did not co-present with a hepatic tumor in any liver sample.

Supplemental Material, Table S6. Logistic regressions of hepatic lesions linked to cellular proliferation on tumor status in mice exposed perinatally to BPA. Odds ratios for hepatic lesions predicted by tumor status in mice exposed perinatally to one of three doses of BPA (control, 50 ng/kg diet, 50 µg/kg diet, or 50 mg/kg diet) were generated using logistic regression models, adjusted for clustering of mice within litters using Generalized Estimating Equations (GEE).

**Hepatic lesion**	**Lesion status**	**Odds ratio (95% CI)**	**Parameter *p*-value**
Oval cell hyperplasia	No neoplastic lesion	Reference	
	Neoplastic lesion	33.95 (12.76, 90.30)	3E-4
Kupffer cell hyperplasia	No neoplastic lesion	Reference	
	Neoplastic lesion	8.75 (3.02, 25.31)	<1E-4
Hepatocyte hypertrophy	No neoplastic lesion	Reference	
	Neoplastic lesion	78.16 (27.02, 226.08)	<1E-4

Neoplastic lesions include hepatocellular carcinomas and hepatic adenomas. Multinucleated hepatocytes may be associated with hepatocyte proliferation but did not co-present with a hepatic tumor in any liver sample.

Supplemental Material, Table S7. SNP genotyping shows mice from this study are 93% C57BL/6J genome-wide, and >99% C57BL/6J on chromosome 1, including the *Hcs7* (*Hepatocellular carcinogenicity locus 7*) locus. Three mice (one yellow A^{vy}/a, one black *a/a* and one C57BL/6J) were genotyped for 74,830 SNPs scattered throughout the genome. Non-C57BL/6J SNPs occur mostly in blocks and may represent low-level introgression from another strain, likely C3H.

Chromosome	Total # SNPs	# Assayable	# SNPs unique to A^{vy}	# SNPs not C57BL/6J	% C57BL/6J	Non-C57BL/6J regions
1	5464	5416	0	6[a]	99.89	
2	5470	5432	54[b]	161	97.04	Agouti locus & tip
3	4458	4416	0	743	83.17	Top two-thirds
4	4408	4341	0	287	93.39	Top quarter and tip
5	4379	4346	0	188	95.67	Lower tip
6	4163	4121	0	733	82.21	Scattered throughout
7	4475	4408	0	380	91.38	Middle third
8	3818	3783	0	7	99.81	
9	3856	3823	0	1381	63.88	Scattered throughout
10	3803	3777	0	5	99.87	
11	4330	4287	0	416	90.30	3 scattered blocks
12	3514	3490	0	3	99.91	
13	3649	3621	0	2	99.94	
14	3235	3205	0	28	99.13	One small block
15	2998	2981	0	138	95.37	One block
16	2868	2849	0	2	99.93	
17	3018	2989	0	1	99.97	
18	2762	2728	0	759	72.18	Scattered on top half
19	2411	2387	0	4	99.83	
X	2378	2342	0	4	99.83	
Y	38	37	0	0	100.00	
Mitochondria	53	51	0	0	100.00	
**Summary**	**75548**	**74830**	**54**	**5247**	**92.99**	**Range is 63.88% to 99.97%**

[a]The *Avy/a* and *a/a* mice differed from the C57BL/6J mouse at only 6 of 5416 SNPs on chromosome 1: B6_rs31362610; B6_rs3659238; B6_01-0749630; B6_rs31375526; UNC010465120; and B6_rs6341208. [b]The *Agouti* (*Avy*) locus is contained within the 54 SNPs on chromosome 2 that differed between yellow and black mice; this is the only region in which the *Avy/a* and *a/a* mice differed.
